# Practical Observations Showing That Mercury Is the Sole Cause of What Are Termed Secondary Symptoms

**Published:** 1839

**Authors:** 


### Practical Observations showing that Mercury is the Sole Cause of what are termed Secondary Symptoms. By *P. J. Murphy*, M.D. Licentiate of the Royal College of Surgeons in Ireland. Churchill, 8vo. pp. 107, 1839.

In his Preface, Dr. Murphy informs us that :—

"In a moral point of view, it may be questioned whether if the doctrines here adduced be true, it would not be better to let them remain untold. This objection carries with it apparently a great deal of weight, and might demand very serious consideration, were it not that we are aware that prostitution exists, and has existed, although it places its votaries beyond the pale of society, is practised under apprehension of a loathsome disease productive of great bodily suffering, and is forbidden by religion.

The advantages and disadvantages, however, of making this doctrine known seem nearly balanced. The dread of contagion being removed, there will be less temptation, on the part of man, to lead those females astray who are already virtuous; and a great amount of physical evil will be avoided by the disuse of a mineral which has destroyed more than it has saved."

This is cheering news for ladies of loose virtue. They will be at a premium so soon as Dr. Murphy's practice takes root. For, as he believes that primary symptoms can be cured without mercury, and that secondary symptoms are only produced by it, it is clear that *mere* pox is no great matter.

The work consists of eleven Chapters. The *first*, is on the identity of the gonorrhœal and syphilitic poisons—the *second*, affirms that syphilis is not a modern disease—the *third* is on chancre—the *fourth*, on bubo—the *fifth*, on ulceration of the throat, palate, nares, &c.—the *sixth*, on iritis—the *seventh* on tophi, nodes, and other affections of the bones—the *eighth*, on venereal swelled testicle—the *ninth*, on syphilis in infants—the *tenth*, on sibbens—and the *last* presents to us the "conclusion."

We shall reverse the ordinary method of reviewing, and notice the last chapter first. We do this that our readers may see at once the gist of Dr. Murphy's observations, and appreciate the objects that he aims at. This is, we conceive, the best method of proceeding in cases of a controversial character, like that of the treatment of syphilis.

The principal doctrines that he advocates are :—

1stly.—That the secretion which produces gonorrhœa is identical with that of chancre.

2ndly.—That consequently, as early an origin must be assigned to chancres and bubo as to gonorrhœa.

3rdly.—That bubo arises from inflammation of a lymphatic vessel, continued to its gland, and not from absorption of a peculiar virus.

4thly.—*That ulceration of the throat, cutaneous eruptions, iritis, nodes, venereal swelled testis, &c., and termed secondary symptoms, are produced solely by mercury.*

5thly.—That chancres and buboes are simple local affections, and consequently not followed by any constitutional symptoms.

6thly.—That the peculiar train of symptoms termed syphilis in infants, have two sources—a mercurialized constitution transmitted by a parent, and sibbens.

7thly.—That sibbens not having been properly discriminated from chancre and mercurial ulcers, is one great cause of the obscurity which surrounds the venereal disease.

We may as well state in limine, *that* the two first positions are extremely doubtful—*that* the third is probably erroneous—*that* the fourth and fifth are totally opposed to our present views of the venereal—and *that* the fifth and sixth are, perhaps, in the same category as the third.

Dr. Murphy enumerates the diseases which he has traced to the carelessness with which mercury is now-a-days exhibited.

1st.—It originates scrofula, transmissible to the offspring.

2nd.—It excites tubercules into action.

3rd.—It leaves the body susceptible of nodes, rheumatism, and other diseases of the fibrous system.

4th.—It produces a tendency to ulceration of the surface and throat.

5th.—Dysentery and ulceration of the intestines.

6th.—A predisposition to aneurism.

7th.—Anterior capsular cataract, as a consequence of iritis.

8th.—Caries of the teeth, with severe pain, mistaken for tic-doloureux.

9th.—A peculiar species of apoplexy.

The only affection, of this formidable list, to which we would allude is the last. We quote the brief observations of our author, because we have seen two cases of a similar description.

"The species of apoplexy which I consider as caused by mercury appears chiefly in young men. I have witnessed four cases; the symptoms were sudden loss of speech, sense and motion, but not so complete as in the ordinary cases; the breathing slightly stertorous; a comatose state for a few days, and then a gradual return of motion, sensation and speech, in the order just mentioned. There was a confusion of intellect for some weeks. The recovery in these four cases was speedy and complete." 106.

We pass to the text from which these conclusions emanate, and which is to be supposed capable of confirming them.

1. *On the Identity of the Gonorrhœal and Syphilitic Poisons*, Dr. Murphy gives some arguments but no facts. It is a question to be decided chiefly by experiment, and no experiments are made. His main battle is this:—

"From my own observations, and from facts recorded by others, I am convinced that the poisons are identical, and that this doctrine would long since have been an axiom in medicine, if the nature of chancre had been properly understood. I have sometimes seen individuals where gonorrhœa, chancres, and bubo were combined, and where the communicating party suffered from one form only of the disease. Ricord says, that in nineteen cases out of twenty of gonorrhœa, he found superficial ulcers of the mucous membrane covering the cervix uteri. This fact, I admit, weakens the inference I wish to draw from the above, when the disease is communicated by the female; but in the reverse there can be no obscurity, if a proper examination be instituted, and the moral character of the female can be depended on. For if a young married woman of irreproachable character is suffering under gonorrhœa, chancres, and bubo, and the husband suffers from gonorrhœa only, which he confesses he has communicated to his wife, I do not see how we can avoid acknowledging their identity, especially if we meet with three or four similar cases. Nor do I perceive anything extraordinary in their combination; for in the conjunctivitis of children we have a purulent discharge from the eye-lids, consequent excoriation and ulceration of the cheek, attended or followed by enlargement, and very frequently suppuration, of the cervical glands, exactly analogous to gonorrhœa, chancre, and bubo." 18.

It will be obvious that the whole plausibility, for proof there clearly is not, of this argument, consists in the vagueness of the term chancre. The name being retained, conveys to our mind the idea of a syphilitic sore with all its peculiar properties. But what evidence is offered of the chancres in the young women being really chancres and not simple ulcerations? And what analogy is there between the *specific* chancre and the excoriation produced by matter running down the cheek? The logician perceives at once the advantage taken of a lax employment of terms.

### On Chancre.

As our author contends that mercury is the sole cause of secondary symptoms, it is not, of course, necessary to distinguish syphilitic from other sores. Nor does he make the attempt. The only observations on this head that we need quote are the following:—

"To Hunter's definition of chancre there can be no valid objection; but when he asserts it will follow the application of the venereal poison only, I must withhold my belief, as from the difference of structure between the glans penis and common tegument, theory would suggest that such could scarcely be the fact, and this has been confirmed to me by observation. On whatsoever part of the body the venereal matter may produce a primary ulcer, nothing like a chancre, as defined by Hunter, is or can be produced, unless on the glans penis, the lips of the mouth, the labia pudendi, the clitoris, and perhaps the nipple; and its corresponding in these parts to this definition is not owing to any peculiarity of cause, but of structure, in which all these parts are more or less analogous.

Ulcers on the glans penis, penetrating three or four lines in depth, and produced by other causes, present the appearance of the Hunterian chancre. It was a circumstance of this kind that first awoke my suspicions that a great deal of obscurity hung over the venereal disease.

A young gentleman showed an ulcer with hardened base and callous edges to a surgeon eminent for his knowledge of this particular disease, who pronounced it chancre, and desired the patient to undergo a mercurial course. On further inquiry it was discovered that it originated from a wound made by the point of a scalpel, which was incautiously carried in his pocket. As the mistake was made by a surgeon of eminence, and as the ulcer answered Hunter's definition, it induced me to investigate more closely his doctrine. I soon learned that an ulcer cannot be pronounced venereal from its appearance alone, and I was highly gratified to find, from Mr. Colles's publication, that he had come to the same conclusion. Had Hunter contented himself with the definition, and not insisted so positively that mercury only was the remedy, the true nature of this simple and trifling disease would long since have been discovered. But the profession relying on his great name, and afraid to delay the specific, lest they should be morally responsible for the deplorable consequences which they believed must be the result, raised up a frightful train of real symptoms while combating a phantom." 35.

We would remark, *first*, that Hunter made a capital blunder, and just what we should expect from his hypothetical mind, in erecting *a* sore as *the* syphilitic;—*secondly*, that we *have* seen venereal ulcers corresponding perfectly with the characters of " chancre " on the skin of the penis, and even on the pubes;—*thirdly*, that the analogy is not very strong between the glans penis and the labia, the mucous membrane commencing indeed on their internal face, but the erectile tissue being scarce developed in them; —and *fourthly*, that although there cannot be doubt of other ulcers than venereal ones, assuming all the external characters formerly supposed *their* exclusive property, yet we should be sorry, on the evidence submitted to us, to affirm that those characters simply depend on the structure of the parts. Indeed a very little consideration shews that it cannot be so.

### ON BUBO.

The opinions entertained by the best informed surgeons on this subject, were expressed, we believe, in an article which we lately published in this Journal. The Hunterian notions on the nature of bubo cannot for an instant be received. They " go the whole hog " in asserting its specific character. It is now generally understood that bubo may be, and generally is, the consequence of the mere extension of inflammatory action, or of " irritation " along the absorbents—or the result of the absorption of a virus. But Dr. Murphy denies the latter, and makes every bubo of the former character. We need not enter on his arguments, for he does not, and he cannot, *prove* his negation.

### ULCERATION OF THE THROAT.

We extract the following as the only observations that appear of material import.

" Hunter describes the true venereal ulcer of the throat ' as a fair loss of substance, part being dug out apparently from the body of the tonsil, possessing a determinate edge, very foul, and having white matter like a slough adhering, which cannot be washed away.' This description, however, is applicable to many other varieties of ulcerated throat, and at present no surgeon of reputation would pronounce an ulceration of the throat to be venereal from its appearance alone. There is no form of the venereal disease in which I have witnessed more hesitation on the part of judicious practitioners in exhibiting mercury than in this. This caution is the result of two circumstances:—First, the impossibility of discriminating the venereal form of ulceration from any other; and, secondly, that the chances are equal of the disease being exasperated instead of benefitted by the exhibition of mercury. Scarlatina, in the adult, when attended with ulceration of the throat, presents appearances almost identical with the definition just quoted from Hunter. When we recollect that there is very little, if any caution employed in prescribing mercury for primary symptoms in scrofulous constitutions—that salivation is aimed at, which is inflammation of the throat and parts contained within the mouth—that parts once inflamed are prone to take on the same action from a trifling cause—that scrofula shews itself frequently as an ulceration of the throat—that it is called into action and its symptoms exasperated by mercury—we need not be surprised if ulceration of the throat should be almost the first of what are termed secondary symptoms. The ulceration, however, is seldom a solitary symptom, being usually accompanied either with cutaneous eruptions, or iritis. It is generally preceded by a febrile state of the system, and may be checked in its progress in its early stage by venesection, tartar emetics, saline diaphoretics, aperients, and regulated diet. When chronic from its commencement, or becoming such from the acute stage, having passed over, cicatrization will seldom fail to follow the use of the vapour bath, the local application of nitrate of silver, either solid or in solution, as a gargle, and the hydriodate of potash in a decoction of sarsaparilla. Warm weather alone is a powerful adjuvant, and many ulcerated throats heal on the approach of summer." 51.

To our experienced readers we need hardly say, *that* ulceration of the throat not unfrequently follows venereal sore, for which *no* mercury has been employed—*that* it often occurs where only a small quantity of mercury has been used—and *that* it not seldom resists all remedies till mercury is given. How this is to be reconciled with the doctrine that it depends upon the use of mercury we do not know. Of course ulceration of the throat often *does* result from abuse of mercury; but it is rushing to the opposite extreme to say that it only occurs from it. The part of the practical surgeon is to discriminate the two orders of cases.

### Tophi, Nodes, and other Affections of the Bones.

"In the whole range of medical science, there is no fact more impressed on my mind, from careful observation, inquiries and study, than that there is not any such disease as a venereal node or any other affection of the osseous system. Mr. S. Cooper, in the last edition of his Surgical Dictionary, refers to the writings of Fallopius, and other eminent physicians, where their opinions are distinctly given, that in lues venerea the bones were never affected unless when mercury had been used. The same opinion is hinted at by several modern authors, but they seem afraid of speaking out plainly, either from not being fully convinced of the fact, or from the doctrine being unpalatable, as it is opposed to the present ideas of the profession." 63.

We cannot subscribe to this unconditional doctrine. We saw a gentleman, a short time ago, who had been treated in Edinburgh for the venereal without mercury. He had, along with tubercular eruptions, thickening of the femur about the great trochanter, attended with all the pain of periosteal inflammation, and with lameness. At the same time we have no hesitation in expressing our opinion that nodes are, in the immense majority of instances, the result of abuse of mercury. This is, we think, as certain as any induction of the sort can be.

Dr. Murphy goes on to observe:—

"The tendency of fibrous structure to disease after a mercurial course is well exemplified by rheumatism. This effect of mercury is now so well known, that it has received a distinct appellation—mercurial rheumatism. I have met with persons satisfactorily tracing this disease to the use of mercury after a lapse of thirty years. The principal argument advanced against mercury being the cause of nodes is, that when exhibited for other diseases than syphilis those symptoms do not follow; and that in India, where mercury is given in so large quantities for hepatitis and dysentery, the disease is never witnessed. That nodes or any other disease of the bones are very unlikely to follow the exhibition of mercury in a climate so hot as India, will be readily admitted. There the intense heat must act as a perpetual vapour bath, and thus carry off all the mineral by perspiration. When we wish to protect our patients from the injurious effect of mercury in this climate, the vapour bath, and whatever else tends to promote perspiration are recommended. It is not very difficult to assign a reason why they do not follow the exhibition of mercury in other diseases. When a patient is placed under a mercurial course for any other disease than syphilis, he is always guarded against the effects of cold; the medicine is given regularly and systematically with a defined object in view, which being obtained, every precaution is then taken by hot baths, purgatives, warm clothing, &c., to prevent injurious effects. Is such the mode adopted when exhibited for syphilis? A scrofulous constitution, which, when affected by any other disease would almost deter us from the exhibition of this potent remedy, is here disregarded. It is administered irregularly, and under every disadvantage. The patient, while pursuing his ordinary avocations, takes his calomel pill night and morning, disregards the weather, pays no attention to diet, while the constant exposure to cold requires that a larger quantity be administered than if he were confined to a warm chamber; and while in other cases the medicine is prescribed in large doses, quickly repeated, here it is taken in small doses at long intervals, until the system is completely mercurialized. To avoid suspicion, the patient neglects the precautions ordinarily employed, and, content with relief from present symptoms, pursues his usual business." 67.

On these observations we would ourselves observe :—*first*, that we do not quite understand how any person could *satisfactorily* trace an attack of rheumatism to mercury taken thirty years before—*secondly*, that hot countries are not so unfavourable to the development of rheumatism, and therefore to the agency of mercury in producing it, as Dr. Murphy supposes; for proof of this we refer to the Army Medical Reports contained in this and our last number—*thirdly*, that we do believe it a pernicious notion to suppose that vapour baths and warm rooms make mercury act more kindly; they tend to bring on a sudden and profuse salivation, and the worst cases that we have witnessed have been those so treated; nay, it was under the old régime, when this very plan was put in practice, that the aggravated ill-consequences of the use of mercury were most conspicuous.

Dr. Murphy quotes Mr. Bacot thus :—

"Mr. Bacot says,—'In the administration of mercury, one precaution is absolutely necessary; either perfect confinement to the house, or such care in the exposure to weather and such precautions against damp clothes, or wet feet, or other sudden transitions of temperature as shall be equal to it. These are old precautions, it is true; so old that I am sorry to say they are worn out; and now we meet with people every day who are under cure of syphilitic complaints, taking a mercurial pill or two, and are all this time pursuing their usual business.'" 67.

We are far from recommending indiscriminate exposure to the weather, nor would we be understood to contend that wet and cold are not bad things. But we think that the open air is made a bug-bear to venereal patients, and that a cure can be effected well enough in the great majority of instances, while patients are pursuing their ordinary avocations. We do not believe, nor have we found, that the administration of mercury is best regulated by the thermometer or the barometer. It's use should be conducted on two great principles, *first*, to maintain the patient's general health—*secondly*, to continue it for that length of time which is found by experience to give most security against secondary symptoms. There are some cases (we find them very few) where confinement during a mercurial course may be adviseable. In the great proportion, such confinement impairs the constitutional powers, and so tends to aggravate the bad effects of mercury. As a matter of fact, we do not find (and we enjoy a fair share of experience) that a *mild and judicious* mercurial course *does* disagree. Few are made worse, and some are even improved by it. Such is the result of our own direct observation. We commenced with no bias towards mercury. So far from that, we were captivated by the specious observations of Carmichael and the statistical facts of the army surgeons. But we enjoyed the acquaintance and witnessed the practice of the late Mr. Rose. Nothing could be more unsatisfactory than the results of the non-mercurial treatment in his hands, and in the latter years of his life Mr. Rose confessed that it was, after all, a delusion. The experience of the Lock Hospital and of private practice established our convictions against the non-mercurial and in favour of the mercurial treatment, and how any practical man can continue a non-mercurialist is, to our minds, incomprehensible.

Dr. Murphy presents us with a chapter on syphilis in infants, to which we are unable to allude. The gist of his remarks is to trace the blotches, &c. of the infant to mercury rather than to syphilis, and, no doubt, he is not very far from the truth.

### On Sibbens.

"Sibbens, or Sivens, is a disease purely local. In the whole range of cutaneous diseases there is none so highly contagious. Its appearance varies according to the structure it attacks; so much so, that in different situations it is described under different names. Fici, crystæ, venereal warts, and condylomata, owe their origin to the same acrimonious secretion which generates sibbens. Even by surgeons of extensive experience and close observation, it is constantly confounded with venereal ulcers, both primary and secondary. Were the signs of this disease generally known, and its treatment properly understood, there is not the least doubt but much of that obscurity which now hangs over the history and treatment of syphilis would instantly be removed.

In the 'Medico-Chirurgical Review,' July, 1834, Mr. Henry Johnson has given us a very accurate description of this disease, as it shows itself when it assumes the form of warts and condylomata, as well as the appearance it presents when situated on the lips, tongue, and tonsils. In the latter form he describes it as *a peculiar superficial whitish ulceration*. His observations I regret have made very little impression, although appearing in a journal which is in the hands of all the reading portion of the profession. Their publication induced me to suppress some remarks on the same disease, which I had ready just then for the press, as I found myself anticipated in almost every one worth making known to the profession." 90.

Sibbens, continues Dr. M., is usually first noticed, especially in females, on the organs of generation, in the form of ulcers, (apparently) raised from the one-eighth to the one-fourth of an inch above the surface; circular when small, oval when large; the diameter varying from the one-fourth of an inch to two inches; surface moist, pale and irregular like a raspberry; free from surrounding inflammation; exuding a fluid, profuse, turbid, adhesive, and peculiarly fætid; firm to the touch, and painless even when rudely pressed. Their usual site is along the convexity and internal surface of the external labia pudendi, the nymphæ and clitoris, the diseased parts, which are naturally in apposition, having corresponding sores; and should there be inattention to cleanliness, the secretion passes to the verge of the anus producing a similar affection (*condyloma*), and if a slight way into the rectum it assumes another form (*ficus*), while in large fat women, the cuticle of whose thighs is frequently abraded by friction, it most usually presents itself under another (*verrucæ*). The inner surface of the vagina may also suffer, and here it assumes quite another appearance, looking like a *white superficial ulceration*, as if the cuticle only had been removed and no further injury done, which is in fact its true pathological character. Owing to anatomical causes, presently to be explained, the disease is less common in the male and not so well marked. The glans penis, and internal surface of the prepuce, the verge of the anus, and the anterior portion of the scrotum, are the parts where it will be usually found. On the two first named parts it is frequently seen under the form of gonorrhœa preputialis, and verrucæ; on the margin of the anus, as fici and condylomata; and on the scrotum it very closely resembles button-scurvy in a moist state. This disease seldom exists long on these parts without producing some affection of the lips, tongue, gums, tonsils, palate or inside of the cheek. Its appearance on these parts, or any mucous surface, is similar to that produced by a fluid when taken too hot.

After some remarks on the communication of this disease to nurses and their recommunication of it to sickly infants, Dr. Murphy goes on to state:—

"The appearance of the so-called ulcers are influenced by several circumstances. Their circular appearance is sometimes lost, and this is owing to two or more coalescing; instead of being raised and rough, they may be smooth and flattened, and this is the appearance they usually shew when situated at the verge of the anus, and is caused by the compression of the opposing surfaces of the nates. We sometimes have them assuming a similar form on the inner surface of the labia from the same cause of pressure. When existing on the scrotum, they may be flattened by the penis when in a depending position. Superficial observers frequently mistake them, when at the margin of the rectum, for rhagazes, or fissures, and this mistake occurs when two or three are partially united, or are in close contact without union. On the labia so much irritation is occasionally excited as to produce in its loose cellular structure considerable œdema. By strict cleanliness or the use of astringent applications, they may cease to secrete, and appear like a small firm tubercle; when those on the inner surface of the labia are in this state they look like a row of flattened beads. The opposed surface of any of these ulcers seldom escapes contamination; and if we examine the anus, labia pudendi, or lower surface of the penis, and find an ulcer, we may expect that unless great attention be paid to cleanliness, the part in contact will certainly be diseased. This is an evident proof that it is contagious in the true sense of the word, and this power of contagion ceases with its power of secretion. When we find the mouth, lips, or tongue affected, it must be from actual contact, and hence the necessity of warning the patient of how necessary cleanliness is, and how cautious he should be in allowing the hand to go near the lips.

Patients in this state are generally set down as labouring under secondary symptoms, and according to the prevailing error of the day, obliged to submit to a mercurial course. The disease of the lips and tonsils will not yield to mercury, hence it is viewed as of a most obstinate nature; an interval is allowed the unfortunate sufferer, the mercury is again resumed; nodes, iritis, and ulcers (the usual effects of this remedy) next display themselves; the symptoms of the disease and the effects of the remedy are combined, the treatment becomes confused, and a correct notion of the disease is supposed to be bestowed on us by the invention of a new word, pseudo-syphilis." 96.

*Treatment.*—For the local "sibbens" Dr. Murphy uses the black-wash as an application. His directions are, to apply it three or four times a-day to the diseased surfaces, by means of common lint, and if practicable oftener; if convenient, the lint, well saturated with the lotion, should be kept on altogether. When the disease exists at the margin of the anus, or on the labia pudendi, the continual application of the lint is indispensable, or otherwise the corresponding surface will inevitably suffer, and thus a new focus of disease will be generated, which will re-act on the original surface. In a few days the advantages of this plan are witnessed, by the condylomata and warts becoming dry and losing their secreting quality; the peculiar fœtid odour ceases, and the parts look dry and clean. Previously to the application of the lotion, the diseased parts should be well washed with soap and water, and this process may be repeated at least once a-day. The astringent lotion is used with the intention of destroying the secreting quality merely, which it does effectually, but its power of removing the tumor (or rather, fungus) is very limited; a very strong solution of the acetate of lead or sulphate of copper destroys them as it destroys the venereal warts, with which they are identical; but as the action of a lotion cannot be restrained to the morbid part, the nitrate of silver in substance will be found the safest, the surest, and the most convenient remedy. It should be freely rubbed over the fungus every second day until every particle is completely destroyed, and the skin appears smooth and healthy. If the slightest remnant escapes, its power of secretion may return, which restores to it an inexhaustible source of contagion. The secreting quality being checked, the tumor may cease to annoy, and a careless patient may desist from remedies. Under these circumstances, the tumor follows the same course as the venereal wart, and may remain for twelve months in a passive state.

For the superficial ulcerations in the mouth, Dr. Murphy uses the nitrate of silver. The surface of the tubercle (for sometimes there are such) should be well rubbed, as well as the neighbouring cuticle, and should be repeated when the black-coloured lamina is detached, until every trace of the morbid product shall be effaced. The black wash should be used in the intervals. The superficial ulcerations of the mouth seem at first to resist the action of the caustic, but on persevering they will be found to lose gradually the white colour, and then to have their diameter lessened.

We have given what appears to us a sufficient account of the views of Dr. Murphy, because we have witnessed such changes in the speculative as well as in the practical departments of medicine, as to make us cautious in clinging to old opinions or rejecting new. But we must in candour confess that Dr. Murphy has not proved his case.

He asserted that mercury was the sole cause of secondary symptoms. He does not account for those instances of such symptoms, which occur (and they are numerous) where no mercury has been employed. He takes indeed the case of sibbens (the confused and objectionable term) and hints that when secondary symptoms occur, and mercury has not been used, it is the sibbens which has given rise to them. But as he adopts Mr. Henry Johnson's description of the venereal condyloma, and admits that it represents what he implies by "sibbens," we protest that we have seen sores of a different character not treated by mercury give rise to secondary symptoms—and we protest also that we have seen secondary symptoms of a different character from those which do follow the venereal condyloma, occur independently of the same calumniated drug.

We speak now from a little acquaintance, or, at all events, attempt at an acquaintance, with venereal disorders. It is strange that complaints so common are generally so ill understood. Few works on the subject are published which do not take a partial, if not erroneous view of them. One author abuses mercury—another will not use it—and a constant see-saw of extravagant theory and contradictory practice is kept up.

For our own parts we do not hesitate to say that the non-mercurial treatment is only a shade better than the ultra mercurial—that the advocates of both are in ruinous extremes—and whoever sets out with the promise of curing syphilis by the one method or the other will, if a candid man, see reasons at last for regretting his mistake, and, if an obstinate man, damage himself and his patients irremediably.

